# Delusion of pregnancy in males and hyperprolactinemia: a case report and review of the literature

**DOI:** 10.1192/j.eurpsy.2025.2204

**Published:** 2025-08-26

**Authors:** I. Besbes, A. Hakiri, C. Bey, K. Mahfoudh, A. Bouallagui, G. Amri, R. Ghachem

**Affiliations:** 1 Department B, Razi hospital, Manouba, Tunisia

## Abstract

**Introduction:**

Delusion of pregnancy is not common among patients suffering from schizophrenia, especially males. The reports in literature regarding this delusion are rare and not conclusive, particularly about its mechanisms. The psychopathology behind this particular condition may involve hormonal and biological ways.

**Objectives:**

Illustrate through a case report and a review of literature the causal relationship between delusion of pregnancy and hyperprolactinemia in a male with schizophrenia.

**Methods:**

This case report involved a comprehensive evaluation of a patient presenting with delusion of pregnancy and hyperprolactinemia. We compared the clinical findings with the existent research after conducting a literature review on different databases such as Pubmed and the national library of medicine using the key words “delusion of pregnancy” “males” and “hyperprolactinemia”

**Results:**

A 30-year-old Tunisian man with a history of schizophrenia who has been of treatment for 6 months, presented to our department with auditory hallucinations, incoherent speech, and a delusion of pregnancy. The patient reported feeling fetal movements and believed a uterus had been implanted in his abdomen after receiving stem cell injections. Since it is the first time that our patient presents this delusion of pregnancy unlike his other relapses, we asked for a prolactinemia test and complete hormonal panel. His prolactin levels were elevated at 59.33 ng/ml (normal range: 15-25 ng/ml), compared to normal baseline levels in previous admissions. The hormonal screening revealed hypogonadotropic hypogonadism. The patient was treated with antipsychotics (haloperidol initially, then switched to clozapine) and sedatives to manage his symptoms and agitation. His prolactin levels were successfully managed with medication, returning to within the normal range (the second measurement being done 8 weeks after the switch to clozapine). The delusion of pregnancy resolved after several weeks of treatment with clozapine. The patient was discharged with ongoing outpatient care to monitor his schizophrenia and prolactin levels.

**Conclusions:**

This case report highlights the association between delusion of pregnancy and hyperprolactinemia in a male patient with schizophrenia. The findings suggest the potential link between these two conditions. Further research is necessary to elucidate the underlying mechanisms and develop evidence-based clinical management.

**Disclosure of Interest:**

None Declared

